# Neutrophil Biomarkers Can Predict Cardiotoxicity of Anthracyclines in Breast Cancer

**DOI:** 10.3390/ijms25179735

**Published:** 2024-09-09

**Authors:** Valentina K. Todorova, Gohar Azhar, Annjanette Stone, Sindhu J. Malapati, Yingni Che, Wei Zhang, Issam Makhoul, Jeanne Y. Wei

**Affiliations:** 1 Division of Hematology/Oncology, Department of Internal Medicine, University of Arkansas for Medical Sciences, Little Rock, AR 72205, USA; 2 Department of Geriatrics, University of Arkansas for Medical Sciences, Little Rock, AR 72205, USA; 3 Winthrop P. Rockefeller Cancer Institute, University of Arkansas for Medical Sciences, Little Rock, AR 72205, USA; 4 Central Arkansas Veterans Healthcare System, Little Rock, AR 72205, USA; annjanette.stone@va.gov; 5 Department of Mathematics and Statistics, University of Arkansas at Little Rock, Little Rock, AR 72205, USA

**Keywords:** anthracyclines, cardiotoxicity, neutrophils, innate immunity, ELISA, RT-qPCR

## Abstract

Doxorubicin (DOX), a commonly used anticancer agent, causes cardiotoxicity that begins with the first dose and may progress to heart failure years after treatment. An inflammatory response associated with neutrophil recruitment has been recognized as a mechanism of DOX-induced cardiotoxicity. This study aimed to validate mRNA expression of the previously identified biomarkers of DOX-induced cardiotoxicity, PGLYRP1, CAMP, MMP9, and CEACAM8, and to assay their protein expression in the peripheral blood of breast cancer patients. Blood samples from 40 breast cancer patients treated with DOX-based chemotherapy were collected before and after the first chemotherapy cycle and > 2 years after treatment. The protein and gene expression of PGLYRP1/Tag7, CAMP/LL37, MMP9/gelatinase B, and CEACAM8/CD66b were determined using ELISA and reverse transcription-quantitative polymerase chain reaction (RT-qPCR). Receiver operating characteristic (ROC) curve analysis was used to determine the diagnostic value of each candidate biomarker. Patients with cardiotoxicity (n = 20) had significantly elevated levels of PGLYRP1, CAMP, MMP9, and CEACAM8 at baseline, after the first dose of DOX-based chemotherapy, and at > 2 years after treatment relative to patients without cardiotoxicity (n = 20). The first dose of DOX induced significantly higher levels of all examined biomarkers in both groups of patients. At > 2 years post treatment, the levels of all but MMP9 dropped below the baseline. There was a good correlation between the expression of mRNA and the target proteins. We demonstrate that circulating levels of PGLYRP1, CAMP, MMP9, and CEACAM8 can predict the cardiotoxicity of DOX. This novel finding may be of value in the early identification of patients at risk for cardiotoxicity.

## 1. Introduction

Cardiotoxic side effects are serious complications of doxorubicin (DOX) chemotherapy that limit its use in high-dose cancer treatment and strongly impact the quality of life and survival of cancer patients [1,2,3,4]. DOX-induced cardiotoxicity can develop immediately or within months or even years after the completion of therapy [5,6]. Acute DOX cardiotoxicity may develop after a single dose or a single course of treatment, and it often remains asymptomatic, presenting with electrocardiographic abnormalities such as QT interval prolongation and left ventricular dysfunction [7,8,9]. These symptoms usually regress; however, they may also progress into chronic, within a year of treatment, or late cardiotoxicity years or decades after treatment [6]. The long-term cardiovascular morbidity and mortality associated with cancer treatment is attributable to complications including asymptomatic left ventricular (LV) dysfunction, congestive heart failure (CHF), pericarditis, myocardial ischemia, arterial hypertension, atrial and ventricular arrhythmias, and thromboembolic disease [9,10,11,12,13]. The chronic cardiotoxicity is considered irreversible, with poor prognosis and limited treatment options [10,11]. Clinical cardiac toxicity has been reported in 6% of patients treated with anthracyclines and subclinical cardiotoxicity in 18% of the patients after follow-up of 9 years [14]. In a case-control study published in 2023 on 2,196 subjects with various cancers treated with DOX, the cumulative incidence of congestive heart failure (CHF) was 7.4% over 15 years, more than 2 times higher than the matched controls [15].

Patients at increased risk of DOX-induced cardiotoxicity are those who received a high cumulative dose (>250 mg/m^2^) [11,16]. Additional factors that increase the risk of cardiotoxicity include history of underlying cardiovascular disease, hypertension, diabetes, obesity, genetic susceptibility, individual differences in drug metabolism, and concomitant exposure to trastuzumab and/or radiation [13,17]. Currently, there is no universal definition for anthracycline-induced cardiotoxicity. The diagnosis is made due to a new-onset heart failure (HF) or imaging evidence of a left ventricular ejection fraction (LVEF) decreased by ≥10% or >15% in LV global longitudinal strain (LVGLS) compared to a previous measurement [11,18,19]. The current diagnostic approach lacks the sensitivity to detect early subclinical cardiac dysfunction and cannot reliably predict future outcomes [18,20,21].

Breast cancer is the most common cancer in women and remains the second leading cause of cancer death among women [22]. DOX-induced cardiotoxicity is particularly important in the growing populations of women afflicted with breast cancer, as anthracyclines are still a major component of the treatment of all subtypes of breast cancer [3,23]. A population-based study of breast cancer survivors showed that women who received anthracyclines, including DOX, daunorubicin, or epirubicin, had higher rates of heart failure than did women who received non-anthracycline or no chemotherapy [24]. The risk of CHD in breast cancer patients is higher at 1 year follow-up and persisting over time, up to 20 years after cancer diagnosis [15]. It was reported that DOX-based therapy of breast cancer patients was associated with more than twice the risk of heart failure compared with controls over 15 years [15].

The mechanism of DOX-induced cardiotoxicity is not completely uncovered, but evidence indicates the involvement of multiple mechanisms, such as oxidative stress, mitochondrial dysfunction, DNA damage via inhibition of topoisomerase 2b (Top2b), dysregulation of calcium homeostasis, apoptosis, and inflammation [13,25,26,27,28,29,30,31]. Recent studies have focused on DOX-induced systemic inflammation and endothelial injury, which can possibly trigger the development and progression of cardiomyopathy [16]. DOX-induced cardiotoxicity is dose-dependent, occurring at low doses with asymptomatic, usually reversible, myocardial injury by the first exposure [32,33,34,35]. The subclinical cardiotoxicity may evolve into irreversible symptomatic chronic cardiotoxicity, leading to heart failure with poor prognosis and limited treatment options years after treatment [8,9,11,12,13,25,36]. As the symptomatic heart failure may occur years after treatment, the early recognition of DOX-induced cardiotoxicity or even the prediction of myocardial injury at early doses of chemotherapy can avoid the permanent cardiac damage and reduce cardiovascular mortality [37]. Several studies, including ours [38], have demonstrated a strong link between DOX-induced inflammation and DOX-induced cardiotoxicity [39,40,41,42]. We have found a correlation between DOX cardiotoxicity and elevated circulating levels of chemokines implicated in inflammatory response and immune trafficking (i.e., CCL23, CCL27, and MIF) [38]. It has been shown that DOX-induced myocardial inflammation is associated with the activation of nuclear factor kappa B (NF-κB) and subsequent release of proinflammatory cytokines implicated in cardiac pathogenesis, such as IL-1β, IL-6, TNF-α, and p38 MAPK [43,44,45]. Our previous data showed that the risk of DOX-induced cardiotoxicity was associated with higher levels of circulating markers of hypercoagulability and inflammation [46,47].

Activated neutrophils release the components of the neutrophil granules, including serine proteases, alpha-defensins, and bactericidal proteins, which in addition to their antimicrobial activity are also involved in several inflammation-associated diseases [48,49,50]. Neutrophil granule proteins are predictive of infarct size, LVEF, new cardiovascular events, and death after acute myocardial infarction [51,52]. Neutrophil infiltration in the heart tissue in response to heart injury resulted in upregulation of inflammatory cytokines [53,54,55,56]. There is currently no standardized, minimally invasive, cost-effective, and clinically verified procedure to monitor cardiotoxicity post anthracycline therapy initiation or to detect the early onset of irreversible cardiovascular complications [57]. Previous data indicate that the early subclinical DOX-induced cardiotoxicity in breast cancer patients is associated with increased expression of neutrophil-specific genes (i.e., PGLYRP1, CAMP, MMP9, CEACAM8, MPO), suggesting that neutrophils and their granular proteins are potential biomarkers for DOX-induced cardiotoxicity. We hypothesize that peripheral blood levels of the neutrophil granular proteins peptidoglycan recognition protein 1 (PGLYRP1), cathelicidin antimicrobial peptide (CAMP), matrix metallopeptidase 9 (MMP9), and carcinoembryonic antigen-related cell adhesion molecule 8 (CEACAM8) are associated with a risk of DOX-induced cardiotoxicity in breast cancer patients. The aim of this study was to validate previously determined mRNA biomarkers of DOX-induced presymptomatic cardiotoxicity, PGLYRP1, CAMP, MMP9, and CEACAM8 and to assay their protein expression in breast cancer patients’ circulation. The changes in expression patterns of these markers and their predictive value for DOX-induced acute cardiotoxicity, as well as the correlation between cardiotoxicity risk and biomarker levels, were also analyzed.

## 2. Results

### 2.1. Demographic Characteristics of the Study Participants

In this study we analyzed peripheral blood samples from 40 patients with early-stage breast cancer treated with DOX-based chemotherapy for protein and mRNA expression of PGLYRP1, CAMP, MMP9 and CEACAM8. We randomly selected 20 patients with DOX-induced abnormal decrease in LVEF ≥10 percentage points after 4 cycles of chemotherapy from baseline or below 50% (CTX) and 20 patients who maintained normal LVEF or a decrease <10% (NCTX). The incidence of subclinical cardiotoxicity in the all patients enrolled in this study resulting from the administration of DOX-based chemotherapy was 12.5%. We also collected and analyzed the expression of these potential biomarkers from all 40 patients at follow-up time after DOX chemotherapy (median months 24.5–48.2) and evaluated the available ECHO, MUGA and/or 12-lead ECG data.

The characteristics of the patients are presented in Table 1. The median age of all patients was between 50 and 52 years. In the CTX group, 13 patients were treated with neoadjuvant DOX chemotherapy and 7 with adjuvant chemotherapy; in the NCTX group 11 patients were treated with neoadjuvant, and 9 with adjuvant chemotherapy. The median change of LVEF versus baseline among the CTX group after the 4th DOX dose was 11.5% points versus 0.2% in the NCTX group. In CTX group 12 patients had hypertension versus 11 patients in NCTX group. Four patients in each group had diabetes, and smoking was reported in 3 patients in CTX versus 4 in NCTX. There were no significant differences between the two groups of patients with respect to the age, BMI, race, history of hypertension, diabetes, smoking and type of breast cancer.

At 2–4 years follow-up, LVEF >20% decrease to less than 40% with signs of heart failure was recorded in 4 patients from the CTX group. Abnormal ECG at 2–4 years follow-up in the CTX group was found in 3 patients with ventricular enlargement and 3 patients with atrial enlargement (Table 1). At the follow-up there were also the following ECG abnormalities: 7 patients with ST and T abnormalities, 4 patients with QT prolongation, and 5 patients with sinus tachycardia in the CTX group, versus 3 patients with ST and T abnormality, 2 patients with QT prolongation and 1 with sinus tachycardia in the NCTX group (Table 1).

None of the patients had neutropenia before the start of the chemotherapy. At 7 days post 1st cycle, one patient from the CTX group had neutropenia (1.09 K/μL) and one patient from the NCTX group showed neutrophilia (13.8 K/μL). At 14 days after the 1st cycle, 2 patients from the NCTX group had neutropenia (0.02 K and 1.02 K), and 2 patients from the CTX group and 1 patient from the NCTX group had elevated neutrophil count (10.84 K and 11.45 K, and 13.20 K respectively). The neutrophil-to-lymphocyte ratio (NLR) increased after the 1st cycle and kept increasing during the subsequent cycles but was not significantly different between the groups (Table 1). 

### 2.2. Analysis of Gene Expression

**Figure 1:**
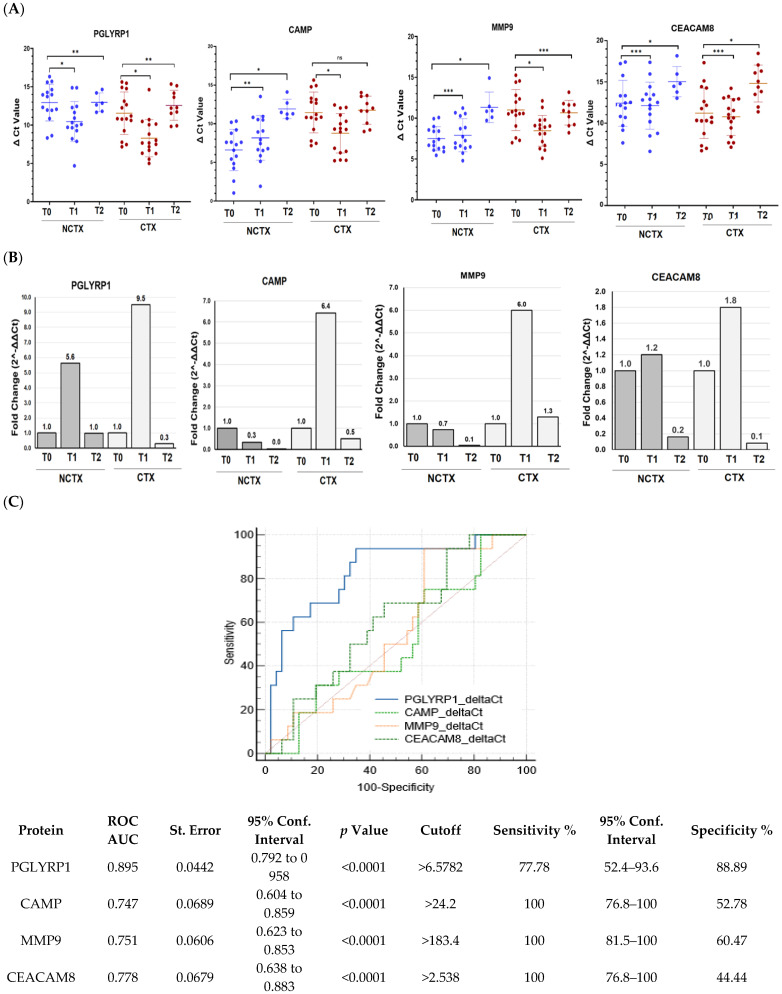
PGLYRP1, CAMP, MMP9, and CEACAM8 mRNA expression levels are increased in the blood of breast cancer patients treated with DOX-based chemotherapy after the 1st cycle (T1) of DOX-based chemotherapy in comparison with the baseline (T0). (**A**) Histogram showing the ΔCt values of PGLYRP1, CAMP, MMP9, and CEACAM8 in PBMCs of patients with cardiotoxicity (n = 16) and patients without cardiotoxicity (n = 16). Each dot represents one sample, and the mean ± SD for each group is indicated. (**B**) Fold change (2^-ΔΔCt) for each of the examined genes comparing T1 and T2, considering the T0 as a calibrator. (**C**) Receiver operating characteristic (ROC) curves for the ability of blood levels of the four mRNAs to predict DOX-induced cardiotoxicity in breast cancer. NCTX, non-cardiotoxicity; CTX, cardiotoxicity; PGLYRP1, peptidoglycan recognition protein 1; CAMP, cathelicidin antimicrobial peptide; MMP9, matrix metallopeptidase; CEACAM8, carcinoembryonic antigen-related cell adhesion molecule 8. *p*-value, * < 0.001, ** < 0.01, *** > 0.1.

We next determined how gene expression of the neutrophil biomarkers PGLYRP1, CAMP, MMP9, and CEACAM8 correlate with the circulating protein levels. The mRNA expression in PBMCs from 32 breast cancer patients, including 16 with DOX-induced cardiotoxicity and 15 patients without, were examined using real-time PCR. The results showed significant upregulation of the gene expression of PGLYRP1, CAMP, MMP9, and CEACAM8 after the initial DOX chemotherapy versus the baseline (T0) in the CTX group of patients (Figure 1). These data validated our previous findings of increased gene expression of leukocyte markers in breast cancer patients with a risk of DOX-induced cardiotoxicity early after the first DOX dose. At 2–4 years post treatment, mRNA levels of PGLYRP1, CAMP, and CEACAM8 decreased to the baseline levels (Figure 1). The ROC curve analysis constructed to compare the relative mRNAs expressions of the examined biomarkers for DOX-induced cardiotoxicity versus non-cardiotoxicity found the following AUCs (Figure 1C, Supplementary Tables S1–S6): PGLYRP1, 0.779 (*p* = 0.001); CAMP, 0.562 (*p* = 0.402); MMP9, 0.610 (*p* = 0.127); and CEACAM8, 0.516 (*p* = 0.806). The cutoff values were PGLYRP1 < 10.45, CAMP < 12.43, MMP9 < 10.03, and CEACAM8 < 14.7 (Supplementary Tables S1–S6).

### 2.3. Analysis of Protein Expression

In this study, we evaluated the potential of the neutrophil biomarkers PGLYRP1/TAG7, CAMP/LL37, MMP9, and CEACAM8/CD66b to predict DOX-induced cardiotoxicity in patients with early-stage breast cancer. We have determined the differences of the circulating biomarkers among patients with abnormal and normal LVEF at diagnosis (baseline), after the first cycle of DOX-based chemotherapy, and >1year after treatment.

**Figure 2:**
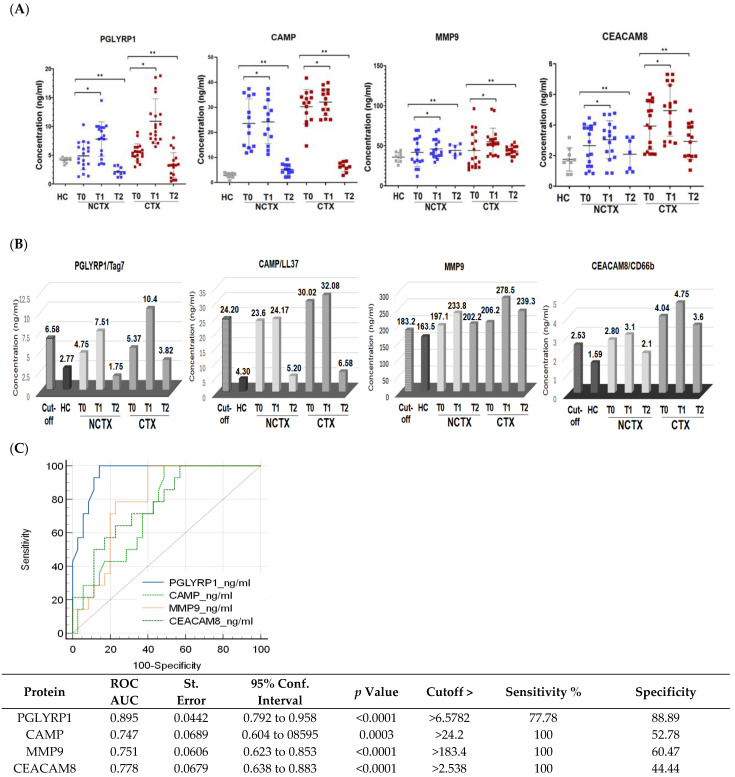
Protein levels of PGLYRP1, CAMP, MMP9, and CEACAM8 are increased in the plasma of breast cancer patients after the 1st chemotherapy cycle and at >1 year after chemotherapy in comparison with the baseline. (**A**) Scatter plots demonstrating the comparison of the protein concentrations of the examined proteins in the plasma of breast cancer patients with cardiotoxicity (CTX) and non-cardiotoxicity (NCTX) at baseline (T0), after the first chemotherapy dose (T1), and at >1 year post treatment (T2). Each dot represents one sample, and the mean and SD for each group is indicated. (**B**) Protein expression levels of the examined markers in comparison with the cutoff values. (**C**) Receiver operating characteristic (ROC) curves for all examined neutrophil markers at CTX_T1 versus HC + NCTX_T0 + CT_T0. NCTX, non-cardiotoxicity; CTX, cardiotoxicity; PGLYRP1, peptidoglycan recognition protein 1; CAMP, cathelicidin antimicrobial peptide; MMP9, matrix metallopeptidase; CEACAM8 carcinoembryonic antigen-related cell adhesion molecule 8. AUC = area under the curve. *p*-Value, * < 0.05, ** > 0.01.

Baseline protein biomarkers: The comparison of the baseline levels (T0) of the examined biomarkers between the two groups of patients using two-sample *t*-test showed significantly (*p* < 0.05) higher plasma concentrations of PGLYRP1, CAMP, MMP9, and CEACAM8 in the CTX group of patients versus the NCTX group (Model 1 in Table 2 and Figure 2). After applying ANCOVA modeling with adjustments for age, race, BMI, diabetes, hypertension, and type of breast cancer, the differences were significant only for PGLYRP1 (Model 2 and Model 3, Table 2).

Protein biomarkers after the first DOX chemotherapy and at > 1 year: A two-sample *t*-test was used to compare the levels of the biomarkers at baseline (T0), after the 1st cycle of DOX-based chemotherapy (T1), and at >2 years after treatment (T2) between the two groups of patients, the cardiotoxicity group (CTX) and non-cardiotoxicity group (NCTX). The results showed significantly higher levels of the examined protein markers at baseline (T0), after the 1st cycle of chemotherapy (T1), and at >2 years after treatment (T2) in the group of patients with cardiotoxicity (CTX) versus the group without (NCTX) (Table 2, Model 1). After adjusting for age, race, BMI, diabetes, hypertension, and type of breast cancer in Model 2 and Model 3, only the levels of PGLYRP1 of the CTX group were significantly different from those of the NCTX (*p* = 0.05) (Model 2 and Model 3, Table 1).

Paired-wise analyses were conducted to determine the changes in the protein markers after the first cycle of chemotherapy (T1) and at >2 years after treatment (T2) in comparison with the baseline (T0) in the two examined groups of patients. In both groups (CTX and NCTX), the plasma levels of PGLYRP1, CAMP, MMP9, and CEACAM8 increased significantly (*p* < 0.05) after the first chemotherapy cycle in comparison with the baseline (T1-T0) (Table 3) and the cutoff value (Figure 2B). At >2 years post chemotherapy (T2), the protein levels of PGLYRP1 and CAMP dropped below T0 (Table 3) and the cutoff value (Figure 2B) in the CTX group, but they were still higher than the levels of the healthy controls (HCs) (Table 2) and the levels in the NCTX group (Figure 2B, Table 3). At this time point (T2), MMP9 also decreased in comparison with T1; it was higher than the baseline (T2-T0), but not significantly (Table 3, Figure 2B).

To assess the diagnostic potential of the four protein biomarker candidates, we used receiver operating characteristic (ROC) curves analysis of the data obtained by ELISA. The ROC curve plots the true positive rate (sensitivity) against the false positive rate (100-specificity). ROC analysis generates an area under the curve (AUC), which is a measure of how well a parameter can distinguish between two diagnostic groups (those with disease/those without disease), where a value of 1 shows perfect performance (sensitivity rises from 0 to 1.0 at 1—specificity of 0, and specificity rises from 0 to 1.0 at sensitivity of 1.0 on an ROC plot). An AUC between 0.7 and 0.9 indicates good diagnostic efficacy. Therefore, the closer the ROC curve is to the upper left corner, the higher the overall accuracy of the test [58]. Youden’s index was used to select the optimal predicted probability cutoff. Using the method suggested by DeLong et al. [59], MedCalc software was used to compare differences in the AUCs. The analysis for each protein biomarker in this study was performed by plotting ELISA data as 0 and 1, where data for “no cardiotoxicity” (HC + NCTX T0 + CTX T0) = 0 and “cardiotoxicity” (CTX T1) =1. The resulting ROC curves are shown in Figure 2C, where the AUCs for PGLYRP1, CAMP, MMP9, and CEACAM8 were 0.895, 0.747, 0.751, and 0.778, respectively, with *p* < 0.001. The cutoff values calculated by the Youden index were 6.57 ng/mL for PGLYRP1, 24.2 ng/mL for CAMP, 183.4 ng/mL for MMP9, and 2.53 ng/mL for CEACAM8 (Figure 2B, Supplementary Tables S1–S6).

**Figure 3:**
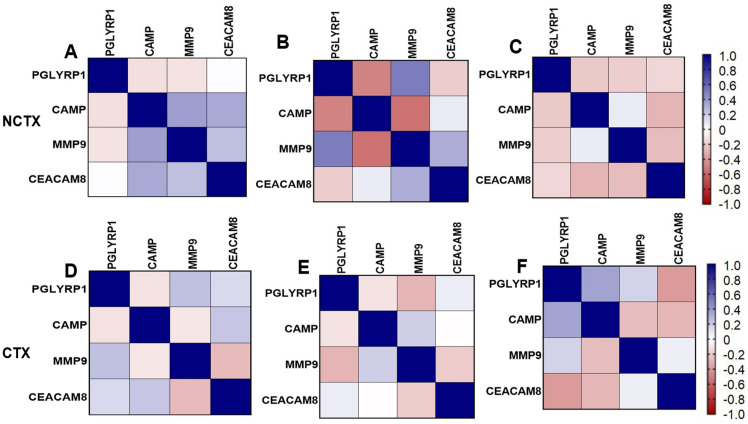
Pairwise Pearson correlations between plasma markers among the study samples by cardiotoxicity and by time points. CTX, cardiotoxicity; NCTX, non-cardiotoxicity. (**A**) NCTX at T0; (**B**) NCTX at T1; (**C**) NCTX at T2; (**D**) CTX at T0; (**E**) CTX at T1; (**F**) CTX at T2.

Correlation matrix of markers: Figure 3 depicts the pairwise Pearson correlations matrix between the plasma markers in the two groups of breast cancer patients at different time points (T0 and T1). From the correlation matrix among NCTX patients at both T0 and T1, PGLYRP1, CAMP, MMP9, and CEACAM8 were positively correlated, reflecting similar modulating pathways for those markers.

## 3. Discussion

The routine approach in monitoring DOX cardiotoxicity includes serial imaging evaluations of the left ventricle function, which provide limited prognostic efficacy as well as limited sensitivity in monitoring early signs of myocardial injury, cardiac stress and the extent of cardiac remodeling [57,60]. Given these limitations as well as poor cost-effectiveness of this approach [57], the present study aimed to identify novel plasma biomarkers in breast cancer patients treated with DOX-based chemotherapy. 

In this study we validated previously determined mRNA biomarkers of DOX-induced cardiotoxicity, PGLYRP1, CAMP, MMP9 and CEACAM8, and demonstrated for the first-time that DOX-based chemotherapy induced elevated circulating levels of their proteins. We found a good correlation between the mRNA expression and the target proteins. The elevated circulating mRNA and protein levels of PGLYRP1/TAG7, CAMP/LL37, MMP9/collagenase IV and CEACAM8/CD66b before and after the first dose of DOX chemotherapy were associated with increased risk for subclinical cardiotoxicity in breast cancer, and the cut-off values were established as predictors for the adverse course of DOX. These findings confirmed our previous data, which suggested that the risk of myocardial injury by the first exposure with DOX in breast cancer may be predicted by the circulating levels of neutrophil-associated proteins PGLYRP1, CAMP, MMP9 and CEACAM8. Taken together, these results indicate that neutrophils through the release of PGLYRP1, CAMP, MMP9 and CEACA8 contribute to DOX-induced cardiotoxicity. These results suggest that targeting these proteins during chemotherapy may decrease the acute DOX-induced cardiotoxicity.

### 3.1. Previous Studies on the Examined Proteins in Cardiovascular Diseases

The four proteins, we selected and studied are of interest in the context of DOX-induced cardiotoxicity for the following reasons. PGLYRP1, a member of the family of antimicrobial proteins, which bind to peptidoglycan (the main component of bacterial cell wall) and play an important role in antibacterial immunity and inflammation [61,62,63,64]. PGLYRP1 is highly expressed predominantly in neutrophils’ and eosinophils’ granules and its expression was associated with various inflammatory conditions [62,65,66,67], including atherosclerotic disease [68,69,70], acute myocardial infarction [71], rheumatoid arthritis [72], asthma [73], oral inflammation [74,75,76], and cancer [77]. Long-term treatment of mice with recombinant PGLYRP1 was associated with both increased atherogenic lesions and reduced fractional shortening of the left ventricle, suggesting that PGLYRP1 may be a potential biomarker for coronary artery disease and heart failure [78]. Cathelicidins, including CRAMP in mouse/rat, and CAMP/LL-37 in humans are a group of antimicrobial peptides (AMPs), which play essential roles in regulating host antibacterial host defense and immunity [79,80,81]. The immunomodulatory functions of cathelicidins have been documented in a variety of inflammatory diseases, such as atherosclerosis [82,83,84], acute coronary syndrome [85], arthritis [86,87], systemic lupus erythematous [88,89] and diabetes [90]. CAMP was found to aggravate inflammation-related myocardial ischemia/reperfusion (MI/R) injury in experimental mice [91]. Matrix metalloproteinases (MMPs) are zinc-dependent endopeptidases secreted by fibroblasts, cardiomyocytes, endothelial cells, and immune cells [92], and their expression is activated by oxidative stress, endothelial dysfunction, and inflammation [93,94,95]. MMPs major function is associated with regulation of extracellular matrix (ECM) turnover and inflammatory signaling [96,97,98], and is involved in in cardiac remodeling and ventricular dilation in heart failure [99]. ECM remodeling plays central role in DOX-induced cardiotoxicity [100,101]. MMP9, collagenase IV is predominantly involved in denaturing basement membrane type IV and V collagens, gelatin, fibronectin and elastin [102], as well as the breakdown of various non-ECM molecules, such as substance P, IL-1β, and myelin basic protein [103]. MMP9 correlated with left ventricular dysfunction after myocardial infarction [104,105], atherosclerosis [106] coronary artery disease [107], and is increased by proinflammatory cytokines, such as interleukin-1 (IL-1) [108], IL-6 [73] and tumor necrosis factor α (TNFα) [74]. CEACAM8 (CD66b) is recognized as a granulocyte activation marker [75], expressed exclusively in humans and two primate species, and absent in mice, and rats [76,77]. CD66-positive neutrophils have been demonstrated in carotid endarterectomy samples from patients with carotid atherosclerosis [109] and patients with psoriasis with an increased risk of cardiovascular diseases [110]. CEACAM8 was one of the plasma proteins related to inflammation identified as key predictive potential biomarkers predictive of heart failure in 3 independent international cohorts [111]. The effect of these biomarkers is mediated and further aggravated by DOX-induced oxidative stress and inflammation specifically in cardiomyocyte, resulting in compromised cardiac structure and left ventricular function decline [93]. Therefore, the panel of circulating biomarkers we have identified in this study presents a strong prognostic utility due to their specific role in DOX- induced cardiotoxicity.

### 3.2. Neutrophils and Role in Inflammation

Neutrophils are early responders to stimuli that lead to tissue injury, including infection, inflammation, trauma, cancer, and thrombosis [112,113,114,115,116]. Neutrophils have been implicated in the progression of cardiovascular diseases including atherosclerosis, thrombosis, and acute coronary syndrome [117,118,119,120,121]. These cells are the first wave of inflammatory cells that infiltrate the injured heart [121], including DOX-induced heart injury [122,123,124,125]. Degranulation of neutrophils releases reactive oxygen species (ROS), antimicrobial peptides, proteases, and neutrophil extracellular traps (NETs), which are all mechanisms for controlling infection/inflammation [126]. Neutrophil granule proteins have detrimental effects in cardiovascular diseases including acute coronary syndrome, thrombosis, and atherosclerosis [113,127,128,129]. We have shown that circulating biomarkers of inflammation, hypercoagulability, and endothelial injury, including C-reactive protein (CRP), thrombomodulin (TM), and thrombin–antithrombin complex (TAT), correlated with the risk of DOX-induced cardiotoxicity in breast cancer patients [41]. DOX is administered into the systemic circulation, and the first contact the drug makes is with the vascular endothelium, inducing oxidative stress, increased apoptosis, and inflammation [47,130], leading to the recruitment of neutrophils and the subsequent release of neutrophil granule proteins [47,130,131]. Studies have shown that blood neutrophils can infiltrate the myocardium and induce cardiac cell damage by the release of chemokines, cytokines, and adhesion molecules [132]. It has been demonstrated that DOX-induced cardiac neutrophil infiltration and the release of MPO [122,123] and S100a8/a9 from their granules contribute to cardiotoxicity [124]. Our earlier work demonstrated that the early inflammatory response induced by DOX chemotherapy was associated with increased levels of myeloperoxidase (MPO), the most abundant protein in neutrophils [46]. Here, we report that DOX therapy induced increased circulating levels of the neutrophil markers PGLYRP1, CAMP, MMP9, and CEACAM8 early after a single dose of chemotherapy in breast cancer patients. Further, we have found that the late DOX-induced cardiotoxicity is associated with elevated levels of MMP9. MMP9 remained elevated in comparison with the baseline and cut-off at >2 years post chemotherapy in the group of patients with cardiotoxicity, suggesting its role in late cardiotoxicity.

Signs of heart failure and LVEF decrease to <40% was recorded in 4 patients with elevated mRNA and protein expression of PGLYRP1, CAMP, MMP9 and CEACAM8 at baseline and after the initial chemotherapy dose. Elevated expression of the examined proteins at >2 years post treatment correlated with several ECG abnormalities, associated with development of cardiomyopathy, including left ventricular and atrial enlargement, ST and T wave abnormality, QT prolongation and sinus tachycardia [133,134,135,136,137,138] at the follow-up in most of the patients with abnormal LVEF. Systemic inflammation induced by DOX is recognized as a mechanism that triggers the development and progression of cardiomyopathy [47,139,140,141]. Several reports indicate that plasma levels of inflammatory markers increased in chronic heart failure (HF) and could also be subclinical indicators of future HF [44]. Inflammation plays a major role in large arteries stiffening, related to atherosclerosis, arteriosclerosis, endothelial dysfunction, smooth muscle cell migration, vascular calcification, increased activity of metalloproteinases, extracellular matrix degradation, oxidative stress, elastolysis, and degradation of collagen [142,143]. Accordingly, it has been demonstrated that anthracyclines induce accelerated vascular aging-like phenotype, including arterial stiffness, which contributes to premature cardiovascular disease in cancer survivors, exposed to these agents [143]. Children who were treated with anthracycline chemotherapy demonstrate greater arterial stiffness when compared with healthy age-matched controls upon follow-up ranging from 1 to 20 years [144,145,146].

In summary, in this study we have evaluated four predictive biomarkers of DOX-induced cardiotoxicity in breast cancer, identified in our previous microarray study for protein expression. We have demonstrated for the first-time that the risk of DOX-induced cardiotoxicity is associated with increased levels of neutrophil-derived granule proteins PGLYRP1, CAMP, MMP9 and CEACAM8 before the start of DOX chemotherapy and after the initial treatment, suggesting their potential as early predictive biomarkers. These findings provide evidence for the efficacy of the proposed biomarkers in predicting cardiotoxicity of DOX before it is identified by MUGA scan or echocardiogram. Higher circulating levels of PGLYRP1, CAMP, MMP9 and CEACAM8 prior to and after the first dose of DOX chemotherapy may further contribute to progressive cardiovascular disorders in breast cancer survivors and may potentially predict the risk of both acute subclinical and late cardiotoxicity. Early identification of the risk for DOX-cardiotoxicity can help in the design of cardioprotective treatments; avoid the accumulating toxic effects from the subsequent doses during treatment and finally to reduce the morbidity and mortality from cardiotoxicity.

The limitations of this study include the relatively small sample size. Further studies with a larger group of patients, along with the dynamic profile of the suggested markers during the course of DOX chemotherapy in correlation with the risk of cardiotoxicity are needed.

## 4. Materials and Methods

### 4.1. Study Population

Women visiting UAMS Winthrop Rockefeller Cancer Institute for diagnosis and treatment of early-stage breast cancer requiring DOX-based chemotherapy were enrolled in this study between 2012 and 2021. Early-stage breast cancer is breast cancer that has not spread beyond the breast or axillary lymph nodes on the same side of the body [147]. This study was approved by the Institutional Review Board (IRB) of UAMS (Protocol #130212) and Central Veterans Healthcare system (CAVHS) IRB (Protocol #1423976-2), where the samples were processed and stored. All patients were treated at the Winthrop Rockefeller Cancer Institute with a predefined protocol which included a combination of DOX (60 mg/m^2^) with cyclophosphamide (600 mg/m^2^) in each cycle for 4 cycles every 2 weeks. All participants signed an IRB approved informed consent in which they were informed in detail about the use of their blood samples and medical records for research purposes. The inclusion criteria included early ER+/PR+/Her2-, ER+/PR-/Her2- or triple negative (ER-/PR-/Her2-), stage I to III breast cancers within 18-99 years of age. Participants were ineligible if they were pregnant, or breast feeding and had no prior history of chemotherapy or radiotherapy. At the time of enrollment, demographic data including age, race, body mass index (BMI) were collected. Baseline data on cardiovascular disease risk factors including the presence of cardiovascular disorders, hypertension, diabetes mellitus and smoking were also collected. A cardiovascular disease score was calculated by counting the number of cardiovascular disease risk factors (hypertension, diabetes mellitus, known coronary artery disease, smoking, vitamin D deficiency or elevated cholesterol). Patients with hypertension who were taking antihypertensive medications (β-blockers and ACE inhibitors) prior to chemotherapy continued with the anti-hypertensive regime concomitant with the DOX-based chemotherapy. Patients with diabetes also continued to be treated with insulin or metformin concomitant with the chemotherapy. Healthy volunteers (n = 10) were also consented and enrolled in the study. Eighty-four patients were enrolled initially in this study, and blood samples were collected, plasma and peripheral blood mononuclear cells (PBMCs) were isolated and stored for further analysis. For this study, plasma and PBMCs of 20 subjects with cardiotoxicity at the end of chemotherapy and 20 subjects without were assayed as described further below. Additionally, 10 healthy women visiting UAMS as family members/caregivers were also consented and enrolled in the study.

### 4.2. Study Endpoints

The aim of this study was to evaluate the predictive value of four neutrophil biomarkers for acute and long-term cardiotoxicity in breast cancer patients treated with DOX-based chemotherapy. Cardiotoxicity was classified as [148,149]: acute cardiotoxicity, immediately after the completion of chemotherapy dose (T1) and late cardiotoxicity which occurs later than one year (2–4 years) after chemotherapy has been completed (T2).

### 4.3. Assessment of Cardiac Function

Cardiac toxicity was evaluated by clinical assessment of LVEF with mixed multigated acquisition (MUGA) scan and/or transthoracic echocardiography (ECHO). DOX-induced cardiotoxicity was defined as LVEF absolute decrease by >10 percentage points in comparison with the baseline (before the chemotherapy) to below 50%, sustained ventricular arrhythmias, or sudden cardiac death [150,151,152]. A standard 12-lead electrocardiography at rest was performed yearly post chemotherapy for monitoring the risk of cardiotoxicity, and data were compared with previously reported results. The 12-lead ECGs parameters evaluated and reported in a number of studies on chemotherapy-induced cardiotoxicity, include markers of LV hypertrophy and left atrial enlargement, such as QRS complex and T-wave, prolongation of PQ, QRS duration, QT interval, QT apex (QTa) interval, and ST [133,134,135,136,153].

However, after the last dose of DOX-based chemotherapy, most of the patients had undergone treatment with taxane and/or chest wall irradiation, which can affect the results obtained 2–4 years after the completion of DOX chemotherapy.

### 4.4. Blood Collection

Blood samples (18 mL) were collected in EDTA collection tubes before the start of DOX-based chemotherapy, 2 weeks after the first cycle and at >1 year after DOX. EDTA-anticoagulated blood was centrifuged at 2000 rpm for 15 min and the top layer containing plasma was removed, and aliquoted. The remaining blood was diluted with an equal volume of phosphate-buffered saline (PBS) without Ca^2+^ and Mg^2+^ and was layered over Ficoll-Paque Plus (GE Healthcare) and was centrifuged at 2000 rpm for 20 min at room temperature in a swinging-bucket rotor without break. The PBMC interface was removed and washed in PBS. The contaminating red blood cells were lysed by incubation for the PBMC pellet with cold ammonium-chloride-potassium (ACK) lysing buffer (GE Healthcare, Wauwatosa, WI, USA) for 10 min, followed by wash with PBS. The plasma and PBMCs were stored at −80 °C.

### 4.5. Neutrophil-to-Lymphocyte Ratio (NLR)

The absolute neutrophil and lymphocyte counts were collected, and the neutrophil-to-lymphocyte ratio (NLR) was evaluated at baseline and 2 weeks after the first DOX chemotherapy dose.

### 4.6. Reverse Transcription-Quantitative Polymerase Chain Reaction (RT-qPCR)

Total RNA was isolated from PBMCs using the RNeasy mini kit (Qiagen, Germantown, MD, USA) according to the manufacturer’s instructions. Concentrations (ng/μL) and OD ratios (260/280 nm) of total RNA were determined using the Nanodrop UV/VIS spectrophotometer (Thermo Fisher, Waltham, MA, USA). RNAs with a A260/A280 ratio between 1.85 and 2.10 were used for cDNA conversion. The quantitative conversion of 800 ng of total RNA to single-stranded cDNA in a 20-μL reaction was performed with High-Capacity cDNA Reverse Transcription Kit (Applied Biosystems, Foster City, CA, USA). Quantitative PCR was performed on 1:10 diluted cDNAs using QuantStudio 12K Flex real-time PCR system software version 1.3 (Applied Biosystems, Watham, MA, USA). Taqman gene expression assays were: Hs00175475_m1 (PGLYRP1), Hs00957562_m1 (MMP9), Hs00189038_m1 (CAMP), Hs00266198_m1 (CEACAM8), Hs99999903_m1 (beta-actin, ACTB) [154]. All quantitative PCRs were performed in a final volume of 10 μL containing 1× of TaqMan Universal PCR Master mix (Applied Biosystems), 1× of each TaqMan Gene Expression Assay (FAM-MGB dyes), and 23 ng cDNA in sterile molecular-grade water. The standard cycling conditions were 50 °C for 2 min, 95 °C for 10 min, followed by 40 cycles of 95 °C for 15 s, and 60 °C for 1 min. Quantitative PCR was performed in triplicate to ensure quantitative accuracy. The results were analyzed using Expression Suite Software version 1.0 (Applied Biosystems). Relative expression levels were calculated for each sample after normalization against the housekeeping genes beta-actin (ACTB) and GTF2B, using the ΔΔCt method for comparing relative fold expression differences [155]. The data are expressed as fold change (FC). Statistical evaluations were done using one-way ANOVA to compare the gene expression levels of sample groups.

### 4.7. Enzyme-Linked Immunosorbent Assays (ELISA)

Stored blood plasma samples were thawed on ice and analyzed using commercially available ELISA kits in duplicate. Quantification of four candidate protein biomarkers was conducted using commercially available ELISA assay kits. We used the following ELISA kits and plasma dilutions: Human PGRPs/PGLYRP1 (Invitrogen# EHPGLYRP1, detection range: 6.14–1500 pg/mL, 1:100 diluted); Human CAMP/LL-37 (MyBioSource, # MBS167379, detection range 0.2–60 ng/mL, undiluted samples), Human CEACAM8/CD66B (MyBioSource, Cat# MBS2024215, detection range 15.62–1000 pg/mL, undiluted samples), Human MMP-9 (R and D Systems, #DMP900, detection range: 0.31–20 ng/mL, 1:100 diluted). ELISA results were read using Synergy H1 microplate reader (BioTek, Winooski, VT, USA). The measured optical densities of the original standards in each of the ELISA kits were used to generated 5-parameter standard curves from which the protein concentrations of the target proteins were interpolated. These were then corrected for dilution factors.

### 4.8. Statistical Analysis

The changes in the blood biomarkers between baseline (T0), after the first DOX dose (T1), and at >1 year post treatment (T2) were determined in association with the risk of DOX-induced cardiotoxicity. The concentration of each protein biomarker in both groups of patients (CTX and NCTX) at each time point (T0, T1, and T2) was presented as means ± SD, and P < 0.05 was considered statistically significant. Two-sample *t*-tests and chi-square (X2)/Fisher exact tests were performed to evaluate differences in patients’ characteristics between groups. Paired *t*-tests, as well as analysis of covariance (ANCOVA) with adjustments for race, age, body mass index (BMI), and type of breast cancer were conducted to determined group differences (CTX vs. NCTX) or paired-wise differences for the patients between different time points (T0-T1 and T2-T0). To compare the difference in fold change between CTX and NCTX groups, two-sample *t*-tests as well as ANCOVA were performed. Analyses were performed using R 4.1.1. Summary descriptive statistics and Pearson correlations were performed using GraphPad Prism version 10.2.3. Normality was assessed using the Kolmogorov–Smirnov test and Shapiro–Wilk test.

Receiver operating characteristic (ROC) curve analysis was used to determine the diagnostic value of each candidate biomarker expression in breast cancer patients with cardiotoxicity (CTX) and non-cardiotoxicity (NCTX) after DOX-based chemotherapy. The performance of each candidate biomarker for discrimination between baseline and one dose of DOX chemotherapy was determined according to ROC “area under the curve” (AUC) values with 95% confidence intervals (95% CI). The cutoff values were determined according to Youden index, and differences in diagnostic performance were analyzed by comparing the ROC curves of MedCalc Software (Version 19.4, Belgium) (www.medcalc.be, accessed on 25 July 2024).

## Figures and Tables

**Table 1:** Demographic characteristics of the study patients.

	Cardiotoxicity (CTX)	Non-Cardiotoxicity (NCTX)	*p*-Value
Age	50.8 ± 10.5	52.5 ± 8.6	0.35
BMI	31.6 ± 7.6	31.7 ± 7.9	0.25
Race			0.14
AA	8	5	
EA	12	15	
Breast cancer			
ER+/PR+/Her2-	15	16	0.58
Triple negative	5	4	0.42
Adjuvant chemotherapy	7	9	
Neoadjuvant chemotherapy	13	11	
Hypertension	12	11	0.84
Diabetes	4	4	
Smoking	3	4	
Neutrophils count			
Before chemotherapy	4.14 ± 1.46	3.73 ± 1.62	0.14
7 days after 1st cycle	3.78 ± 1.98	4.92 ± 3.01	0.15
14 days after 1st cycle	4.80 ± 3.21	4.27 ± 3.13	0.29
Neutrophil-to lymphocyte ratio (NLR)			
Before chemotherapy	2.26 ± 1.42	2.44 ± 1.15	0.34
7 days after 1st cycle	2.68 ± 2.03	4.02 ± 3.22	0.07
14 days after 1st cycle	4.10 ± 3.42	4.18 ± 2.82	0.47
LVEF baseline	67.8 ± 6.1	61.4 ± 4.6	0.0006
LVEF after 4 DOX cycles	56.3 ± 8.6	61.2 ± 5.7	0.02
LVEF 2–4 years post DOX	54.3 ± 11.5	65	N/A
ECG changes at >1 year post chemotherapy			N/A
Left ventricle enlargement	3		
Left atrial enlargement	3	1	
ST and T wave abnormality	7	3	
QT prolongation	4	2	
Sinus tachycardia	5	1	
Heart failure	4		

All data are presented as means ± standard deviation (SD). *p*-values represent differences in NCTX vs. CTX patients for each characteristic. Continuous variables were evaluated by two-sample *t*-tests (italic), and chi square (X^2^) tests were used to investigate the differences in distributions of categorical variables from the groups. AA, African American; EA, European American; ER, estrogen receptor; PR, progesterone receptor; Her2, human epidermal growth factor receptor 2; LVEF, left ventricle ejection fraction; ECG, electrocardiogram. Data significant at *p* < 0.05.

**Table 2:** Differences in the circulating protein biomarkers among patients with cardiotoxicity and no cardiotoxicity at baseline and after the first cycle of DOX-based chemotherapy.

	HC	CTX	NCTX	Mean Difference	^a^ Model 1			^b^ Model 2		^c^ Model 3	
		Mean ± SD		(CTX-NCTX)	*p*	FC	Trend	*p*	FC	*p*	FC
	Baseline										
PGLYRP1(ng/mL)	2.8 ± 0.4	5.3 ± 1.7	4.75 ± 2.4	0.62	0.047	1.13	ABN ↑	0.05	1.48	0.05	1.52
CAMP (ng/mL)	4.3 ± 3.6	30.2 ± 6.8	23.58 ± 5.8	6.7	0.02	1.29	ABN ↑	NS	1.2	NS	1.45
MMP9 (ng/mL)	163.5 ± 29.5	206.2 ± 30.8	197.1 ± 84.4	9.1	0.03	1.05	ABN ↑	NS	1.2	NS	1.33
CEACAM8 (ng/mL)	1.60 ± 0.4	4.04 ± 1.45	2.80 ± 1.5	1.24	0.02	1.19	ABN ↑	NS	1.2	NS	1.38
	After 1st cycle										
PGLYRP1(ng/mL)		10.3 ± 4.6	7.51 ± 3.3	2.8	0.003	1.38	ABN ↑	NS	1.62	NS	1.24
CAMP (ng/mL)		32.08 ± 5.30	24.17 ± 8.67	7.9	0.03	1.33	ABN ↑	NS	1.89	NS	1.69
MMP9 (ng/mL)		278.6 ± 94.7	233.8 ± 66.2	44.8	0.05	1.19	ABN ↑	NS	1.89	NS	1.3
CEACAM8 (ng/mL)		4.75 ± 1.53	3.07 ± 1.85	1.68	0.02	1.55	ABN ↑	NS	1.74	NS	1.44
	>2 years after treatment										
PGLYRP1(ng/mL)		3.82 ± 2.0	1.7 ± 1.1	2.01	0.03	2.47	ABN ↓	NS	1.12	NS	1.08
CAMP (ng/mL)		6.58 ± 2.11	5.2 ± 2.29	1.37	0.07	1.11	ABN ↑	NS	1.30	NS	1.28
MMP9 (ng/mL)		239.6 ± 28.9	202.2 ± 38.8	37.45	0.01	1.18	ABN ↑	NS	1.24	NS	1.50
CEACAM8 (ng/mL)		3.6 ± 0.7	2.1 ± 0.3	1.4	0.04	1.10	ABN ↑	NS	1.14	NS	1.11

^a^ Two-sample *t*-tests comparing CTX and NCTX. ^b^ ANCOVA comparing CTX and NCTX adjusted for race, age, and BMI. ^c^ ANCOVA comparing CTX and NCTX adjusted for race, age, BMI, hypertension, diabetes, and type of breast cancer. Data significant at *p* < 0.05.

**Table 3:** Comparison between the means of after–before (T1-T0) and (T2-T0) DOX-based chemotherapy differences in the two groups of breast cancer patients, the group with cardiotoxicity (CTX) and the group with no cardiotoxicity (NCTX).

	CTX									NCTX								
	Mean						Mean			Mean						Mean		FC
	T0	T1	T2	T1-T0	P	FC	T2-T0	P	FC	T0	T1	T2	T1-T0	P	FC	T2-T0	P	FC
PGLYRP1 (ng/mL)	5.3	10.3	3.8	5.0	0.003	1.9	−1.1	0.5	0.7	4.1	7.5	1.7	3.4	0.004	1.8	−0.7	0.3	0.85
CAMP (ng/mL))	30.2	32.1	6.6	1.9	0.02	1.0	−22	0.6	0.2	23.5	24.2	5.2	0.5	0.04	0.1	−18.3	0.4	0.22
MMP9 (ng/mL)	206.2	278.6	239.6	72.4	0.04	1.3	7.5	0.4	1.0	208.8	239.6	202.2	30.8	0.03	1.1	−6.61	0.5	0.9
CEACAM8 (ng/mL)	4.0	4.7	3.5	0.7	0.02	1.2	−0.5	0.2	0.8	2.7	3.6	2.9	0.9	0.05	1.3	0.2	0.1	0.3

Paired *t*-tests comparing T0 and T1 and comparing T0 and T2. CTX, patients with DOX-induced cardiotoxicity; NCTX, patients with no cardiotoxicity; MeanDiff, difference between the means of T1-T0 and T2-T0 of the group of patients.

## Data Availability

The data generated from this study are available upon reasonable request.

## Supplementary Materials

The following supporting information can be downloaded at: https://www.mdpi.com/article/10.3390/ijms25179735/s1.
